# Nitric oxide involvement in the acrosome reaction triggered by leptin in pig sperm

**DOI:** 10.1186/1477-7827-9-133

**Published:** 2011-10-04

**Authors:** Saveria Aquila, Francesca Giordano, Carmela Guido, Vittoria Rago, Amalia Carpino

**Affiliations:** 1 Department of Pharmaco-Biology, University of Calabria 87036 Arcavacata di Rende (Cosenza), Italy; 2 Department of Cell Biology, Faculty of Pharmacy, University of Calabria 87036 Arcavacata di Rende (Cosenza), Italy; 3 Centro Sanitario. University of Calabria 87036 Arcavacata di Rende (Cosenza) Italy

**Keywords:** acrosome reaction, leptin, nitric oxide, NOSs, pig spermatozoa

## Abstract

**Background:**

Nitric oxide (NO) is a signaling molecule produced by intracellular nitric oxide synthase (NOS) enzymes. This free radical appears to affect sperm capacitation, a maturation step preceding acrosome reaction. Recent studies have reported leptin ability to promote capacitation and acrosome reaction in pig male gametes.

**Methods:**

This study has investigated nitric oxide production in leptin-treated pig spermatozoa by fluorescence-activated cell sorting, while the intracellular NOS isoforms were assessed by Western blot analysis. In addition, acrosome status of treated-spermatozoa was evaluated by FITC-PNA staining.

**Results:**

Significant increases of nitric oxide levels and acrosome reaction extent were detected in leptin-treated spermatozoa, but both the effects were reversed in presence of *L*-NAME. Furthermore, the immunoblots of sperm extracts have evidenced three bands of ~160 Kd(bNOS), ~130 Kd (iNOS) and ~135 Kd (eNOS).

**Conclusions:**

The identification of the three intracellular NOS isoforms suggests that pig spermatozoa could produce NO, while the augmented nitric oxide levels in leptin-treated male gametes indicates the capacity of the hormone to induce nitric oxide production. Furthermore, the inhibitory effect of *L*-NAME and of Ab-ObR on the promotion of acrosome reaction triggered by leptin suggests a possible involvement of NO in the hormone action.

## Background

The role of metabolic substances in the mechanisms controlling reproductive processes is emerging in the last years. Leptin is mainly known as a hormonal link between energy stores and energy homeostasis but it appears to be also involved in reproductive activity [1]. In the pig, leptin regulates reproductive functions at hypothalamus-pituitary level [2], but our recent works have also revealed its role in the control of peripheral structures. In fact, we have showed the expression of leptin and its receptor in pig testicular and epididymal tissues [3] as well as in pig spermatozoa [4]. Furthermore, we evidenced leptin capacity to affect pig sperm survival and capacitation. Capacitation is a crucial step of mammalian sperm maturation which induces biochemical and biophysical changes in sperm membrane, leading to a specialized exocytosis known as acrosome reaction [5]. Capacitation and acrosome reaction are two complex processes which appear to be controlled by cross-talks between different pathways [6-9] including the nitric oxide-dependent pathway [10,11]. Nitric oxide (NO) is a highly reactive signaling molecule, synthesized by intracellular NO-synthase (NOS) enzymes [12], which appears to regulate different sperm functions [13,14].

The aim of the present study was to investigate the capacity of pig spermatozoa to produce NO in response to leptin treatment. Therefore, the expression of intracellular NOS enzymes has been also assessed and the possible involvement of NO in acrosome reaction triggered by leptin has been evaluated.

## Methods

### Chemicals and antibodies

*Chemicals *Laemmli sample buffer, pre-stained molecular weight marker, Percoll (colloidal PVP coated silica for cell separation), Earle's balanced salt solution, propidium iodide, fluorescein isothiocyanate-labeled peanut (Arachis hypogaea) agglutinin (FITC-PNA) and all other chemicals were purchased from Sigma Chemical (Milan, Italy). Porcine leptin was purchased from Protein Laboratories Rehovot (Rehovot, Israel), acrylamide bisacrylamide was from Labtek Eurobio (Milan, Italy). Triton X-100, ECL Plus Western blotting detection system, Hybond™ECL™, were purchased from Amersham Pharmacia Biotech (Buckinghamshire, UK). Fluorescent probe 4,5-diaminofluorescein-2/diacetate (DAF 2/DA) and *N*-nitro-*L*-arginine methyl ester (L-NAME) were from Vinci Biochem,(Firenze, Italy)

*Antibodies *Mouse monoclonal anti-nitric oxide synthase inducible (iNOS) (N-9657), anti-nitric oxide synthase brain (bNOS)(N-2280) and anti-nitric oxide synthase endothelial (eNOS) (N-9532) were from Sigma Chemical (Milan, Italy). Polyclonal rabbit anti OBR(H-300), peroxidase-coupled anti-mouse IgG and rabbit polyclonal anti β-actin were from Santa Cruz Biotechnology (Heidelberg, Germany).

### Animals and semen samples

The investigation has been conducted on semen from 6 fertile male pigs (*Sus scrofa domestica*, Large White) kept at " Swine Artificial Insemination Centre " (Rende, Cosenza, Italy). The animals were 22 to 28 month-old and their weights were from 260 to 300 kg. Individual fresh ejaculates were collected by the gloved hand method and filtered immediately by Universal Semen bags (Minitub, Tiefenbech, Germany). Semen was transported within half an hour to the laboratory, it was diluted 1:10 with TBS buffer and centrifuged on a discontinuous Percoll density gradient (72%/90%) to remove bacteria and debris [15].

### Sample treatments

Percoll-purified spermatozoa were incubated with Earle's medium (uncapacitating medium) for 30 minutes at 39°C and 5% CO_2 _without or with 10 nM leptin, 0.7 mM *L*-NAME. Some cells were also pre-treated (15 min) with the anti-OBR Ab (autocrine blockage). The dose of leptin was chosen on the basis of our previous findings [4].

### NO detection

Intracellular NO was measured as previously described [16]. Briefly, leptin-treated spermatozoa were loaded with DAF-2/DA (10 μM) and incubated (120 min, 37°C) in the dark. Some of the samples were loaded with the NOS inhibitor, *L*-NAME (0.7 mM), 30 min prior to DAF-2/DA loading. Care was taken to prevent exposure to light throughout the rest of the experiment as the probe is light-sensitive. After incubation with DAF-2/DA the cells were analyzed by fluorescence-activated cell sorting (FACS analyzer) (excitation wavelength 488 nm and emission wavelength 530 nm) at a single-cell level and data were analyzed using Cell Quest software (Becton Dickinson, NJ, USA). The mean fluorescence intensity of the analyzed sperm cells was determined after gating the cell population by forward and side scatter signals. In total, 25000 events were acquired, but non-sperm particles and debris were excluded by prior gating, thereby limiting undesired effects on overall fluorescence. The final gated populations usually consisted of 15 000-20 000 sperm cells.

The experiments were repeated at least four times for each sample.

### Western blot analysis

Western blot analysis was used to identify NOS enzymes in sperm samples. Percolled spermatozoa were washed twice with Earle's balanced salt solution and then were centrifuged for 5 min at 5000 × g. The pellet was resuspended in lysis buffer as previously described [17]. Equal amounts of proteins (80 μg) were boiled for 5 min, separated by 10% polyacrylamide gel electrophoresis, transferred to nitrocellulose sheets and probed with an appropriate dilution of anti-iNOS (1:1000), anti-bNOS (1:3000) and anti-eNOS (1:3000) antibodies. The bound of the secondary antibody was revealed with the ECL Plus WB detection system according to the manufacturer's instructions. Negative controls were prepared using tissue lysates, where antigens were previously removed by pre-incubation with specific antibodies (1 hour at room temperature) and subsequently immunoprecipitated with protein A/G-agarose. β-actin served as a control for equal loading. The experiments were repeated at least four times for each sample.

Human breast cancer cells, MCF7, expressing the three NOS isoforms, were used as control. MCF7, obtained from American Type Culture Collection (ATCC), were cultured in a 25 cm^2 ^cell culture flask containing DMEM supplemented with 10% FBS and 10 units of penicillin and 10 μg/ml of streptomycin, at 37°C, in a humidified incubator containing 5% CO_2_.

### Acrosome reaction

Spermatozoa incubated with leptin, combined or not with *L*-NAME or Ab-OBR, were re-suspended in unsupplemented Earle's medium (Earle's balanced salt solution without Ca, Mg, Phenol red and NaHCO_3_) (5 × 10^6 ^spermatozoa/ml), were placed in a conical tube and cultured for 2 hours in an atmosphere of 5% CO_2 _in air at 39°C. Spermatozoa incubated only with Earle's medium were used as control. Then acrosomal status was monitored using the acrosome-specific fluorochrome fluorescein isothiocyanate-labeled peanut (Arachis hypogaea) agglutinin (FITC-PNA) in conjunction with DNA-specific fluorochrome propidium iodide (PI) as a viability test [18]. Briefly, sperm suspension (1 × 10^6 ^ml) was exposed to FITC-PNA (10 μg/ml) and propidium iodide (12 μmol/l) for 5 minutes at 39°C and then fixed by adding 1 ml of 12.5% (w/v) paraformaldehyde in 0.5 mol Tris/l (pH 7.4). The slides were immediately examined with an epifluorescence microscope (Olympus BX41) with a multiple fluorescence filter (U-DM-DA/FI/TX2) observing a minimum of 200 spermatozoa per slide (100× objective). Acrosomal status was assessed according to the staining patterns.

#### Staining patterns

Spermatozoa with a nuclear red PI staining were considered as dead cells while sperm cells without PI staining were considered as live cells.

Live spermatozoa were classified in 2 main categories on the basis of the FITC-PNA staining as follows: i) acrosome-reacted cells with uniform green FITC-PNA fluorescence of acrosome cap ii) acrosome-intact cells without any fluorescence. Values were expressed as percentage. Four replicate experiments were performed for each semen sample.

### Statistical analysis

Data, presented as mean ± SEM, were evaluated by the one-way analysis of variance (ANOVA). The differences in mean values were calculated at a significance level of *P *≤ 0.05. The Wilcoxson test was used after ANOVA as post hoc test.

## Results

### NO production by pig spermatozoa

**Figure 1:**
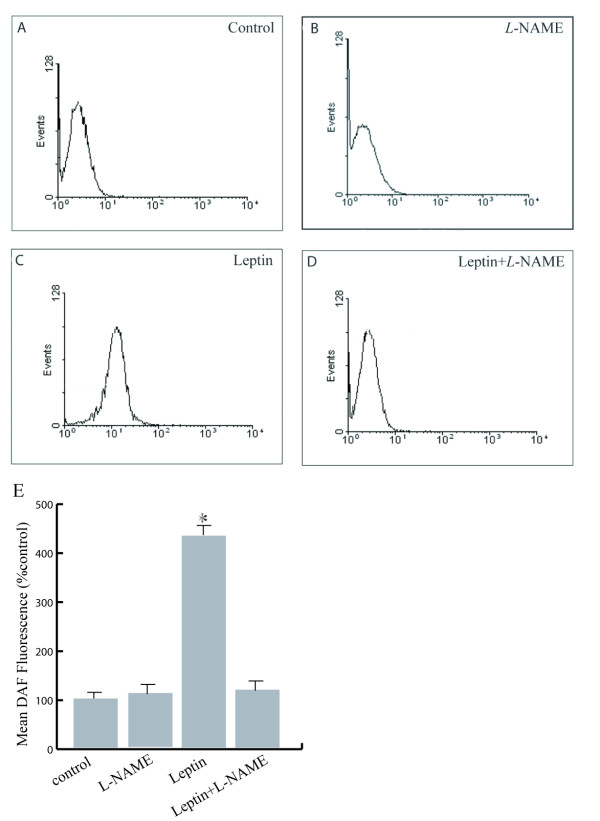
**Representative histograms of DAF fluorescence in pig sperm**. (**A**): Control sperm. (**B**):spermatozoa incubated with *L*-NAME. (**C**): leptin-treated sperm.(**D): **leptin-treated sperm in presence of *L*-NAME. (**E**): data above reported which are expressed as mean fluorescence (percentage of control, control adjusted to 100%). Values are mean percentage ± SD. (* p < 0.05).

Incubation of pig spermatozoa with 10 nM leptin induced a significant increase of intracellular NO levels (Figure 1B) with respect to control sperm (Figure 1A), while pre-treatment of spermatozoa with the NOS inhibitor, *L*-NAME, reversed the leptin effect (Figure 1C). The DAF-2/DA fluorescence data are also expressed as mean fluorescence (percentage of control, control adjusted to 100%) (Figure 1D)

### Western blot analysis

**Figure 2:**
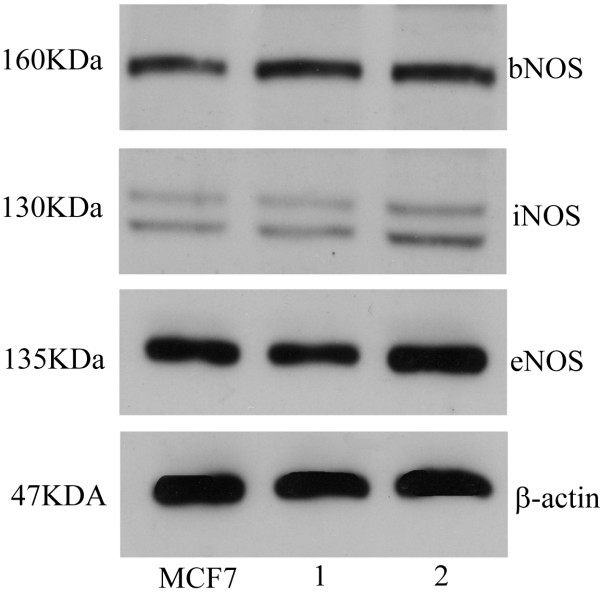
**Immunoblots of nitric oxide synthase (NOS) isoforms from pig sperm extract: **positive control in *lanes *MCF7; representative pig sperm samples in *lanes *1 and 2. Numbers on the left-hand side correspond to molecular weights of detected proteins, while specific NOS isoforms are indicated on the right-hand side. β-Actin serves as a loading control.

The immunoblots of sperm extracts showed three NOS bands: ~160 kDa, ~130 kDa and ~135 kDa corresponding to bNOS, iNOS and eNOS respectively (Figure 2: *lanes *1 and 2). Three NOS bands at the same mobility were observed in the positive control (Figure 2: *lane *MCF7). The negative controls were unlabelled.

### Acrosome reaction in leptin-treated pig spermatozoa

**Figure 3:**
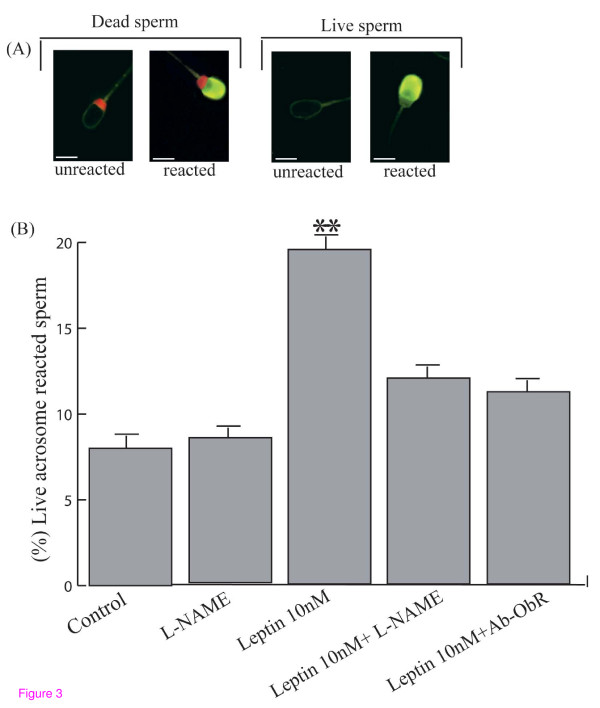
**Acrosome reaction in leptin-treated pig sperm**. (**A**) Representative FITC-PNA fluorescence pattern of spermatozoa incubated with 10 nM leptin. *Scale bars*: 5 μm. (**B**) Incidence of positive acrosome reaction in control spermatozoa, spermatozoa incubated with *L*-NAME, spermatozoa incubated with 10 nM leptin, spermatozoa incubated with 10 nM leptin in presence of *L*-NAME and spermatozoa incubated with 10 nM leptin+ Ab-ObR. Values are mean percentage ± SD (** p < 0.01).

Figure 3A shows a representative fluorescence pattern of pig spermatozoa stained with FITC-PNA + PI for the assessment of acrosome status and sperm viability, after leptin treatment. No significant difference was observed in the incidence of dead spermatozoa between treated-spermatozoa and control sperm.

A higher percentage of acrosome-reacted cells (FITC-PNA positive cells) was detected in 10 nM Lep-treated spermatozoa (19 ± 2%) with respect to control spermatozoa (8 ± 3%), while *L*-NAME reversed the acrosome reaction extent triggered by leptin (12 ± 3%) as well as Ab-OBR (11 ± 3%) (Figure 3B)

## Discussion

Low and controlled concentrations of nitric oxide play an important role in sperm physiology, while excessive NO levels are detrimental [19].

Nitric oxide(NO) is a free radical involved in the intra- and intercellular signaling mechanisms. NO is generated from the oxidation of L-arginine to L-citrulline by 3 isoforms of NADPH-dependent NO synthases (NOS) [12]. Two constitutive Ca^2+^- dependent isoforms are known, i.e. neuronal or brain NOS (n/bNOS) firstly found in neurons and endothelial NOS (eNOS) firstly found in endothelial cells [20]. In addition, one inducible Ca^2+^-independent isoform (iNOS) has been also described [21], firstly identified in macrophages.

The NOS-NO system appears to be implicated in different events leading to acquisition of fertilizing ability. The NOS isoforms were detected in different mammalian spermatozoa such as in mouse [22], in bull [23] and in human spermatozoa [24,25], while NO was able to affect sperm motility, capacitation and acrosome reaction in the mouse [26], in the hamster [27], in the bull [28,29] and also in the human [30-35].

Concerning porcine spermatozoa, NOS enzymes were immunolocalized in male testicular germ cells [36] and NOS isoforms were detected in testis extract [37] but their expression in ejaculated spermatozoa is still unknown. However, NO appears to play a role in functional activity of the male gamete. In fact, it has been reported that *L*-arginine and geldamycin (a heat shock protein 90-specific inhibitor) are able to induce capacitation and acrosome reaction in boar spermatozoa, trough the NO signal pathway [18,38].

Recent studies by our own and others have revealed that leptin and insulin, two hormones linked to energy homeostasis, can be involved in porcine sperm biology [4,39]. Particularly, we have detected leptin and its receptor in pig spermatozoa, localizing them at acrosomal level, and have evidenced the hormone ability to promote capacitation and acrosome reaction [4].

The present study has been addressed to investigate if NO signalling could mediate leptin promotion of pig acrosome reaction. On the basis of our previous findings [4] we have selected the dose of leptin (10 nm leptin) inducing the best stimulatory response on pig sperm capacitation. In addition, to better investigate the effect of leptin on intracellular NO production, we have used uncapacitated spermatozoa (such as in our previous work) avoiding the interference of the high NO levels generated by spermatozoa in capacitating condition [32]

Our investigation has identified, for the first time, the three NOS isoforms (bNOS, eNOS and iNOS) in pig ejaculated spermatozoa indicating the potential ability of the male gamete to synthesize NO. Then, we have shown a significant increase of intracellular NO production in leptin-treated spermatozoa, suggesting that NO could mediate the hormone action. Therefore, we have detected sperm acrosome reaction induced by leptin in presence or not of a NOS inhibitor (*L*-NAME) and of Ab-ObR to block leptin from binding to its specific receptors.

As expected, leptin was able to promote acrosome reaction but the NOS inhibitor and AB-ObR reversed the effect of the hormone. These results suggest the implication of the NOS-NO system in the promoter action of leptin on acrosome reaction of pig sperm. A similar result has been recently reported in human spermatozoa [40].

In our previous work [4] we investigated the main transduction pathways regulated by leptin in pig spermatozoa suggesting that extracellular signal-regulated kinase (ERK)1/2 and phosphatidylinositol Phosphate Kinase 3 (PI3K)/Akt are implicated in the hormone action. In fact, leptin, through its receptor, was able to activate positively ERK1/2 as well as Akt. Because it has been reported that ERK1/2 and PI3K/Akt can regulate NOS activity, via serine phosphorylation [41-43], it is reasonable to hypothesize that the signalling cascade, induced by leptin, could cause NOS activation in pig spermatozoa leading to intracellular NO increase. Furthermore, NOS activation is also calcium-dependent, therefore it can be speculated that intracellular calcium stores could be mobilized by leptin in pig male gametes for the synthesis of NO.

## Conclusions

The identification of the three intracellular NOS isoforms suggests that pig spermatozoa could produce NO, while the augmented nitric oxide levels in leptin- treated male gametes indicates the capacity of the hormone to induce nitric oxide production. Furthermore, the inhibitory effect of *L*-NAME and of Ab-ObR on the promotion of acrosome reaction triggered by leptin suggests a possible involvement of NO in the hormone action.

## Competing interests

The authors declare that they have no competing interests.

## Authors' contributions

SA the author responsible for performing the experiments and participating in the analysis and interpretation of data.

FG carried out the FACS experiments.

CG carried out the pig sperm treatment and WB analysis.

VR carried out the acosome reaction experiment.

AC the author responsible for conception, design, analysis and interpretation of data as well as of drafting manuscript. All authors read and approved the final manuscript.

## References

[B1] Barash IA Cheung CC Weigle DS Ren H Kabigting EB Kuijper JL Clifton DK Steiner RA Leptin is a metabolic signal to the reproductive system Endocrinology 1996 137 3144 3147 10.1210/en.137.7.3144 8770941

[B2] Caprio M Fabbrini E Isidori AM Aversa A Fabbri A Leptin in reproduction Trends Endocrinol Metab 2001 12 65 72 10.1016/S1043-2760(00)00352-0 11167124

[B3] Rago V Aquila S Guido C Carpino A Leptin and its receptor are expressed in the testis and in the epididymis of young and adult pigs Anat Rec (Hoboken) 2009 292 736 745 10.1002/ar.20880 19306434

[B4] Aquila S Rago V Guido C Casaburi I Zupo S Carpino A Leptin and leptin receptor in pig spermatozoa: evidence of their involvement in sperm capacitation and survival Reproduction 2008 136 1 23 32 10.1530/REP-07-0304 18367502

[B5] Bedford MJ Cross NL Knobil E, Neill JD Sperm capacitationencyclopedia of reproduction 1999 4 Academic, San Diego 597 602

[B6] de Lamirande E Leclerc P Gagnon C Capacitation as a regulatory event that primes spermatozoa for the acrosome reaction and fertilization Mol Hum Reprod 1997 3 175 94 10.1093/molehr/3.3.175 9237244

[B7] Leclerc P de Lamirande E Gagnon C Interaction between Ca2+, cyclic 3',5' adenosine monophosphate, the superoxide anion, and tyrosine phosphorylation pathways in the regulation of human sperm capacitation J Androl 1998 19 434 443 9733146

[B8] Bailey JL Factors regulating sperm capacitation Syst Biol Reprod Med 2010 56 5 334 48 10.3109/19396368.2010.512377 20849222

[B9] Fraser LR The "switching on" of mammalian spermatozoa: molecular events involved in promotion and regulation of capacitation Mol Reprod Dev 2010 77 3 197 208 19908247 10.1002/mrd.21124

[B10] Zhang H Zhou QM Li XD Zhao WP Wang YL Liu H Li N Ginsenoside Re promotes human sperm capacitation through nitric oxide-dependent pathway Mol Reprod Dev 2007 74 497 501 10.1002/mrd.20583 17013883

[B11] De Lamirande E Lamothe G Reactive oxygen-induced reactive oxygen formation during human sperm Free Radic Biol Med 2009 46 4 502 510 10.1016/j.freeradbiomed.2008.11.004 19071212

[B12] Palmer R M Ashton DS Moncada S Vascular endothelial cells synthesize nitric oxide from L-arginine Nature 1988 333 664 666 10.1038/333664a0 3131684

[B13] Herrero MB Gagnon C Nitric Oxide: a novel mediatore of sperm function J Androl 2001 22 3 349 356 11330633

[B14] Roessner C Paasch U Glander HJ Grunewald S Activity of nitric oxide synthase in mature and immature human spermatozoa Andrologia 2010 42 2 132 7 10.1111/j.1439-0272.2009.01005.x 20384805

[B15] Kuster CE Hess RA Althouse, Immunofluorescence reveals ubiquitination of retained distal cytoplasmic droplet on ejaculated porcine spermatozoa J Androl 2004 25 340 347 15064310 10.1002/j.1939-4640.2004.tb02798.x

[B16] Lampiao F Strijdom H du Plessis SS Direct nitric oxide measurement in human spermatozoa: flow cytometric analysis using the fluorescent probe, diaminofluorescein Int J Androl 2006 29 564 567 10.1111/j.1365-2605.2006.00695.x 16968498

[B17] Aquila S Sisci D Gentile ME Middea E Siciliano L Andò S Human ejaculated spermatozoa contain active P450 aromatase J Clin Endocrinol Metab 2002 87 3385 3390 10.1210/jc.87.7.3385 12107254

[B18] Funahashi H Induction of capacitation and acrosome reaction of boar spermatozoa by L-arginine and nitric oxide synthesis associated with the anion transport system Reproduction 2002 124 857 864 10.1530/rep.0.1240857 12530923

[B19] Herrero MB de Lamirande E Gagnon C Nitric oxide is a signaling molecule in spermatozoa Curr Pharm Des 2003 9 5 419 425 10.2174/1381612033391720 12570819

[B20] Knowles RG Moncada S Nitric oxide synthases in mammals Biochem J 1994 298 249 58 7510950 10.1042/bj2980249 PMC1137932

[B21] Förstermann U Closs EI Pollock JS Nakane M Schwarz P Gath I Kleinert H Nitric oxide synthase isozymes. Characterization, purification, molecular cloning, and functions Hypertension 1994 23 1121 1131 7515853 10.1161/01.hyp.23.6.1121

[B22] Herrero MB Goin JC Canteros MG Franchi AM Perez Martinez S Polak JM Viggiano JM F Gimeno MA The nitric oxide synthase of mouse spermatozoa FEBS Lett 1997 411 39 42 10.1016/S0014-5793(97)00570-X 9247138

[B23] Meiser H Schulz R Detection and localization of two constitutive NOS isoforms in bull spermatozoa Anat Histol Embryol 2003 32 321 325 10.1111/j.1439-0264.2003.00459.x 14651478

[B24] Herrero MB Perez Martinez S Viggiano JM Polak JM Gimeno MAF Localization by indirect immunofluorescence of nitric oxide synthase in mouse and human spermatozoa Reprod Fertil Dev 1996 6 931 934 10.1071/rd9960931 8876053

[B25] O'Bryan MK Zini A Cheng CY Schlegel PN Human sperm endothelial nitric oxide synthase expression: correlation with sperm motility Fertil Steril 1998 70 6 1143 1147 10.1016/S0015-0282(98)00382-3 9848308

[B26] Herrero MB Cebral E Boquet M Viggiano JM Vitullo A Gimeno MA Effect of nitric oxide on mouse sperm hyperactivation Acta Physiol Pharmacol Ther Latinoam 1994 44 3 65 69 7663015

[B27] Kameshwari DB Siva AB Shivaji S Inhibition of in vitro capacitation of hamster spermatozoa by nitric oxide synthase inhibitors Cell Mol Biol 2003 49 421 428 12887095

[B28] Rodriguez PC O'Flaherty CM Beconi MT Beorlegui NB Nitric oxide induces acrosome reaction in cryopreserved bovine spermatozoa Andrologia 2005 37 5 166 172 10.1111/j.1439-0272.2005.00674.x 16266394

[B29] Rodriguez PC Valdez LB Zaobornyj T Boveris A Beconi MT Nitric Oxide and superoxide anion production during Heparin-Induced capacitation in cryopreserved bovine spermatozoa Reprod Dom Anim 2011 46 1 74 81 10.1111/j.1439-0531.2010.01583.x 20149138

[B30] Lewis SEM Donnelly ET Sterling ELS Kennedy MS Thompson W Chakravarthy U Nitric oxide synthase and nitrite production in human spermatozoa: evidence that endogenous nitric oxide is beneficial to sperm motility Mol Hum Reprod 1996 2 873 878 10.1093/molehr/2.11.873 9237228

[B31] Herrero MB de Lamirande E Gagnon C Nitric oxide regulates human sperm capacitation and protein-tyrosine phosphorylation in vitro Biol Reprod 1999 61 3 575 581 10.1095/biolreprod61.3.575 10456831

[B32] Herrero MB Chaatterjee S Lefievre L de Lamirande E Gagnon C Nitric oxide interacts with the cAMP pathway to modulate capacitation of human spermatozoa Free Radic Biol Med 2000 29 522 536 10.1016/S0891-5849(00)00339-7 11025196

[B33] Revelli A Costamagna C Moffa F Aldieri E Ochetti S Bosia A Massobrio M Lindblom B Ghigo D Signaling pathway of nitric oxide-induced acrosome reaction in human spermatozoa Biol Reprod 2001 64 1708 1712 10.1095/biolreprod64.6.1708 11369599

[B34] Zhang H Zhou QM Li XD Duan X Min FL Liu B Yuan ZG Ginsenoside Re increases fertile and asthenozoospermic infertile human sperm motility by induction of nitric oxide synthase Arch Pharm Res 2006 29 145 151 10.1007/BF02974276 16526279

[B35] Miraglia E De Angelis F Gazzano E Hassanpour H Bertagna A Aldieri E Revelli A Ghigo D Nitric oxide stimulates human sperm motility via activation of the cyclic GMP/protein kinase G signaling pathway Reproduction 2011 141 47 54 10.1530/REP-10-0151 20965947

[B36] Kim HC Byun JS Lee T K Jeong CW Ahn M Shin T Expression of nitric oxide synthase isoforms in the testes of pigs Anat Histol Embryol 2007 36 135 138 10.1111/j.1439-0264.2006.00739.x 17371387

[B37] Ambrosino A Russo D Lamanna C Assisi L Rizzo M Vittoria A Cecio A Isoforms of nitric oxide synthase in the pig testis Acta Vet BRNO 2003 72 493 498

[B38] Hou ML Huang SY Lai YK Lee WC Geldanamycin augments nitric oxide production and promotes capacitation in boar spermatozoa Anim Reprod Sci 2008 104 56 68 10.1016/j.anireprosci.2007.01.006 17280805

[B39] Carpino A Rago V Guido C Casaburi I Aquila S Insulin and IR-beta in pig spermatozoa: a role of the hormone in the acquisition of fertilizing ability Int J Androl 2010 33 3 554 562 19531084 10.1111/j.1365-2605.2009.00971.x

[B40] Lampiao F Du Plessis SS Insulin and Leptin enhance human sperm motility, acrosome reaction and nitric oxide production Asian J Androl 2008 10 799 807 10.1111/j.1745-7262.2008.00421.x 18645684

[B41] Cai H Li Z Davis ME Kanner W Harrison DG Dudley SC Akt-dependent phosphorylation od serine^1179 ^and mitogen-activated protein kinase kinase/extracellular signal-regulated kinase1/2 cooperatively mediate activation of the endothelial nitric-oxide synthase by hydrogen peroxide Mol Pharmacol 2003 63 325 331 10.1124/mol.63.2.325 12527803

[B42] Lorenz M Wessler S Follmann E Michaelis W Düsterhöft T Baumann G Stangl K Stangl V A constituent of green tea, epigallocatechin-3-gallate, activates endothelial nitric oxide synthase by a phosphatidylinositol-3-OH-kinase-cAMP-dependent protein kinase-, and Akt-dependent pathway and leads to endothelial-dependent vasorelaxation J Biol Chem 2004 279 7 6190 6195 14645258 10.1074/jbc.M309114200

[B43] Chen CC Ke WH Ceng LH Hsieh CW Wung BS Calcium and phosphatidynolositol 3-kinase/Akt-dependent activation of endothelian nitric oxide synthase by apigenin Life Sci 2010 87 743 749 10.1016/j.lfs.2010.10.014 21034748

